# Supplementary material for: Incidence and progression of diabetic retinopathy and blindness in Indonesian adults with type 2 diabetes
Source: PLoS One. 2025 Aug 29;20(8):e0322093. doi: 10.1371/journal.pone.0322093 (PMC12396671; doi:10.1371/journal.pone.0322093)
Supplement: S1 Table — (DOCX) [file pone.0322093.s001.docx]

Supplementary table 1. Diabetic Retinopathy Incidence, VTDR Incidence, and DR Progression by other risk factors

| Risk Factors | DR Incidence | | | | | VTDR Incidence | | | | | DR Progression | | | | |
| --- | --- | --- | --- | --- | --- | --- | --- | --- | --- | --- | --- | --- | --- | --- | --- |
|  | At Risk | Incidence rate  (95% CI) | HR  (95% CI) | P^1^ | P^2^ | At Risk | Incidence rate  (95% CI) | HR (95% CI) | P^1^ | P^2^ | At Risk | Progression rate  (95% CI) | HR  (95% CI) | P^1^ | P^2^ |
| Total subject | 377 | 34.6  (29.3-40.8) | - | - | - | 507 | 24.5  (20.2-29.8) | - | - | - | 318 | 35.1  (29.8-41.3) | - | - | - |
| Fasting glucose (per 10 mg/dL) | 377 | - | 1.01  (0.96-1.04) | 0.15 | 0.06 | 507 | - | 1.02  (0.98-1.03) | 0.21 | 0.41 | 318 | - | 1.01  (0.99-1.03) | 0.31 | 0.47 |
| Systolic (mmHg) |  |  |  |  |  |  |  |  |  |  |  |  |  |  |  |
| <140 | 239 | 37.3  (30.4-45.9) | Reference | - | - | 318 | 24.9  (19.3-32.1) | Reference |  |  | 176 | 33.2  (26.7-41.3) | Reference |  |  |
| 140 – 170 | 116 | 29.5  (21.9-39.8) | 0.85  (0.59-1.21) | 0.36 | 0.49 | 164 | 24.7  (17.8-34.3) | 1.08  (0.71-1.63) | 0.72 | 0.53 | 130 | 40.5  (31.4-52.3) | 1.32  (0.95-1.84) | 0.11 | 0.11 |
| >170 | 22 | 37.7  (18.9-75.3) | 0.88  (0.46-1.71) | 0.72 | 0.62 | 25 | 18.84  (7.07-50.2) | 0.67  (0.26-1.71) | 0.40 | 0.21 | 12 | 18.8  (7.07-50.2) | 0.52  (0.19-1.44) | 0.21 | 0.17 |
| Diastolic (mmHg) |  |  |  |  |  |  |  |  |  |  |  |  |  |  |  |
| <80 | 84 | 40.8  (28.7-58.0) | Reference |  |  | 104 | 21.1  (12.9-34.4) | Reference |  |  | 51 | 27.6  (18.0-42.4) | Reference |  |  |
| 80 – 90 | 267 | 33.6  (27.6-40.8) | 0.68  (0.46-1.02) | 0.06 | 0.13 | 370 | 24.9  (19.9-31.3) | 0.93  (0.54-1.60) | 0.80 | 0.96 | 246 | 37.6  (31.2-45.2) | 1.13  (0.70-1.82) | 0.61 | 0.66 |
| >90 | 26 | 28.8  (15.0-55.4) | 0.62  (0.32-1.22) | 0.17 | 0.17 | 33 | 28.8  (15.0-55.4) | 1.16  (0.55-1.48) | 0.70 | 0.69 | 21 | 28.8  (15.0-55.4) | 0.94  (0.42-2.06) | 0.87 | 0.82 |
| BMI |  |  |  |  |  |  |  |  |  |  |  |  |  |  |  |
| Under – normal | 123 | 27.1  (20.1-36.7) | Reference |  |  | 173 | 25.8  (18.9-35.2) | Reference |  |  | 132 | 41.9  (32.9-53.5) | Reference |  |  |
| Over | 161 | 36.8  (28.6-47.3) | 1.44  (0.98-2.12) | 0.06 | 0.06 | 219 | 26.5  (19.7-35.7) | 1.10  (0.72-1.67) | 0.66 | 0.59 | 121 | 31.9  (24.4-41.9) | 0.80(0.56-1.14) | 0.20 | 0.21 |
| Obese | 93 | 43.5  (31.6-59.7) | 1.75  (1.14-2.68) | **0.010** | **0.011** | 115 | 18.3  (11.2-29.8) | 0.79  (0.44-1.40) | 0.42 | 0.37 | 65 | 28.6  (19.3-42.3) | 0.75  (0.48-1.18) | 0.22 | 0.24 |
| Education |  |  |  |  |  |  |  |  |  |  |  |  |  |  |  |
| None | 36 | 28.6  (16.6-49.3) | Reference |  |  | 52 | 26.4  (15.0-46.6) | Reference |  |  | 38 | 41.9  (26.7-65.6) | Reference |  |  |
| Primary | 125 | 28.5  (20.8-39.1) | 1.04  (0.56-1.92) | 0.91 | 0.85 | 162 | 16.1  (10.6-24.4) | 0.65  (0.33-1.30) | 0.22 | 0.38 | 106 | 32.2  (23.9-43.3) | 0.82  (0.46-1.43) | 0.48 | 0.63 |
| Junior HS | 171 | 39.28(31.08-49.66) | 1.63(0.90-2.93) | 0.10 | 0.13 | 234 | 28.62 (21.75-37.66) | 1.33 (0.712.50) | 0.37 | 0.55 | 140 | 35.36 (27.62-45.26) | 1.01-0.59-1.73) | 0.98 | 0.99 |
| Senior HS | 41 | 36.2  (22.2-59.1) | 1.48  (0.72-3.04) | 0.29 | 0.25 | 53 | 27.1  (15.4-47.8) | 1.26  (0.57-2.77) | 0.56 | 0.49 | 32 | 33.9  (20.5-56.3) | 0.98  (0.50-1.93) | 0.95 | 0.98 |
| Smoking Status |  |  |  |  |  |  |  |  |  |  |  |  |  |  |  |
| No smoker | 277 | 36.1  (29.9-43.6) | Reference |  |  | 369 | 24.6  (19.6-30.9) | Reference |  |  | 231 | 35.1  (28.9-42.5) | Reference |  |  |
| Active | 35 | 29.7  (16.9-52.4) | 0.81  (0.43-1.52) | 0.52 | 0.59 | 51 | 27.3  (15.1-49.3) | 1.08  (0.56-2.10) | 0.82 | 0.71 | 33 | 34.7  (20.6-58.6) | 0.98  (0.58-1.66) | 0.96 | 0.96 |
| Ex-smoker | 62 | 30.1  (19.4-46.6) | 0.88  (0.55-1.40) | 0.58 | 0.58 | 83 | 24.1  (14.7-39.3) | 1.02  (0.59-1.74) | 0.96 | 0.89 | 52 | 36.1  (24.2-53.9) | 1.07  (0.69-1.64) | 0.76 | 0.87 |
| Medication |  |  |  |  |  |  |  |  |  |  |  |  |  |  |  |
| No Medication | 16 | 40.7  (18.3-90.5) | Reference |  |  | 17 | 27.1  (10.2-72.2) | Reference |  |  | 8 | 27.1  (10.2-72.2) | Reference |  |  |
| Oral Medication | 307 | 34.8  (28.9-41.8) | 1.00  (0.46-2.19) | 0.99 | 0.72 | 416 | 23.4  (18.7-29.3) | 0.97  (0.35-2.69) | 0.95 | 0.63 | 249 | 32.3  (26.7-39.1) | 1.30  (0.49-3.43) | 0.59 | 0.82 |
| Oral +Insulin | 50 | 31.8  (20.8-48.8) | 0.94  (0.39-2.25) | 0.89 | 0.46 | 70 | 30.3  (19.6-46.9) | 1.29  (0.43-3.85) | 0.64 | 0.71 | 60 | 50.0  (35.6-70.4) | 2.11  (0.77-5.76) | 0.15 | 0.36 |
| Stroke and CVD |  |  |  |  |  |  |  |  |  |  |  |  |  |  |  |
| No | 357 | 34.4  (29.0-40.8) | Reference | - | - | 481 | 24.6  (20.1-30.1) | Reference |  |  | 304 | 35.7  (30.2-42.2) | Reference |  |  |
| Yes | 17 | 36.7  (17.5-76.9) | 1.03  (0.50-2.15) | 0.93 | 0.78 | 22 | 26.2  (10.9-62.9) | 1.06  (0.48-2.32) | 0.89 | 0.36 | 13 | 20.9  (7.86-55.8) | 0.59  (0.24-1.45) | 0.25 | 0.10 |
| Gangrene |  |  |  |  |  |  |  |  |  |  |  |  |  |  |  |
| No | 361 | 34.9  (29.5-41.4) | Reference |  |  | 483 | 23.9  (19.5-29.4) | Reference |  |  | 285 | 33.1  (27.8-39.4) | Reference |  |  |
| Yes | 13 | 27.9  (13.3-58.5) | 1.00  (0.49-2.02) | 0.99 | 0.73 | 20 | 35.9  (18.7-68.9) | 1.99  (1.04-3.82) | 0.03 | 0.15 | 32 | 63.7  (39.0-104) | 2.54  (1.49-4.35) | **0.001** | **0.002** |
| Neuropathy |  |  |  |  |  |  |  |  |  |  |  |  |  |  |  |
| No | 199 | 37.5  (29.7-47.3) | Reference |  |  | 258 | 24.8  (18.6-33.0) | Reference |  |  | 126 | 27.9  (21.4-36.6) | Reference |  |  |
| Yes | 175 | 31.9  (25.2-40.4) | 0.88  (0.64-1.23) | 0.47 | 0.32 | 245 | 24.5  (18.7-32.1) | 1.00  (0.68-1.48) | 0.98 | 0.66 | 191 | 41.2  (33.5-50.7) | 1.50  (1.07-2.10) | **0.018** | **0.044** |
| Residence |  |  |  |  |  |  |  |  |  |  |  |  |  |  |  |
| Rural | 242 | 29.9  (24.1-37.1) | reference |  |  | 326 | 20.78  (16.0-26.9) | Reference |  |  | 220 | 34.6  (28.3-42.3) | reference |  |  |
| Urban | 135 | 44.2  (34.2-57.0) | 1.34  (0.96-1.90) | 0.08 | 0.07 | 181 | 32.2  (23.9-43.4) | 1.35  (0.90-2.04) | 0.15 | 0.13 | 98 | 35.9  (27.1-47.6) | 0.88  (0.48-1.25) | 0.47 | 0.34 |

P^1^: crude

P^2^: multivariate regression model adjusted by age, gender, diabetes duration, systolic blood pressure, fasting blood glucose, gangrene, body mass index (BMI), diabetes medication, and residential area.

Abbreviations: BMI, body mass index; CVD, cardiovascular diseases; DM, diabetes mellitus; DR, diabetic retinopathy; HS, high school; HR, hazard ratio; VTDR, vision threatening diabetic retinopathy
