# Supplementary material for: Incidence and progression of diabetic retinopathy and blindness in Indonesian adults with type 2 diabetes
Source: PLoS One. 2025 Aug 29;20(8):e0322093. doi: 10.1371/journal.pone.0322093 (PMC12396671; doi:10.1371/journal.pone.0322093)
Supplement: S2 Table — (DOCX) [file pone.0322093.s002.docx]

Supplementary table 2. Incidence rate of blindness

| Risk Factor | At Risk | Incidence rate/ 1000 person-year (95% CI) | Hazard Ratio (crude) | P-value 1 (crude) | P-value 2 (Multivariate) |
| --- | --- | --- | --- | --- | --- |
| Overall | 520 | 8.33 (5.95 – 11.7) |  |  |  |
| Gender % |  |  |  |  |  |
| Male | 143 | 7.70 (5.07 – 11.7) | Reference |  |  |
| Female | 377 | 9.80 (5.56 – 17.3) | 1.24 (0.62 – 2.51) | 0.542 | 0.593 |
| Age at DM Diagnosis |  |  |  |  |  |
| <40 | 67 | 11.7 (5.26 – 26.1) | Reference |  |  |
| 40 – 50 | 187 | 7.80 (4.32 – 14.1) | 0.68 (0.25 – 1.88) | 0.459 | 0.514 |
| 50 – 60 | 182 | 7.73 (4.28 – 13.9) | 0.67 (0.24 – 1.84) | 0.438 | 0.533 |
| 60 – 70 | 69 | 8.71 (3.63 – 20.9) | 0.77 (0.23 – 2.52) | 0.662 | 0.734 |
| >70 | 15 | 6.27 (0.88 – 44.5) | 0.58 (0.07 – 5.01) | 0.625 | 0.672 |
| Diabetes duration |  |  |  |  |  |
| Per 10 years increase | 520 | - | 1.22 (0.77-1.91) | 0.397 | 0.376 |
| 0 – 4 years | 274 | 6.38 (3.70 – 10.9) | Reference |  |  |
| 5 – 10 years | 162 | 10.4 (6.06 – 17.9) | 1.64 (0.75 – 3.52) | 0.221 | 0.204 |
| >10 years | 84 | 10.0 (5.02 – 20.06) | 1.34 (0.55 – 3.28) | 0.513 | 0.504 |
| Fasting glucose (per 10 mg/dL) | 520 | - | 1.01 (0.95 – 1.04) | 0.893 | 0.981 |
| Systolic Blood Pressure (mmHg) |  |  |  |  |  |
| <140 | 310 | 7.05 (4.38 – 11.3) | Reference |  |  |
| 140-170 | 185 | 10.3 (6.21 – 17.1) | 1.50 (0.75 – 3.02) | 0.251 | 0.243 |
| >170 | 25 | 9.41 (2.36 – 37.7) | 1.19 (0.28 – 5.05) | 0.812 | 0.830 |
| Diastolic Blood Pressure (mmHg) |  |  |  |  |  |
| <80 | 105 | 7.90 (3.55 – 17.6) | Reference |  |  |
| 80-90 | 379 | 7.98 (5.35 – 11.9) | 0.88(0.36-2.18) | 0.781 | 0.725 |
| >90 | 36 | 12.8 (4.81 – 34.1) | 1.49 (0.41 – 5.46) | 0.546 | 0.581 |
| BMI |  |  |  |  |  |
| Under/normal weight | 182 | 10.9 (6.82 – 17.7) | Reference |  |  |
| Overweight | 219 | 6.64 (3.68 – 11.9) | 0.62(0.29-1.32) | 0.215 | 0.228 |
| Obese | 119 | 6.86 (3.08 – 15.3) | 0.69(0.27-1.74) | 0.432 | 0.475 |
| Education Level (%)* |  |  |  |  |  |
| No School | 52 | 11.0 (4.58 – 26.5) | Reference |  |  |
| Elementary School | 170 | 9.51 (5.52 – 16.4) | 0.93 (0.32 – 2.67) | 0.888 | 0.940 |
| Junior High School | 238 | 7.86 (4.65 – 13.3) | 0.84 (0.29 – 2.41) | 0.743 | 0.653 |
| Senior High School | 56 | 4.52 (1.13 – 18.1) | 0.48 (0.09 – 2.50) | 0.382 | 0.362 |
| Smoking Status |  |  |  |  |  |
| Not a Smoker | 385 | 9.78 (6.80 – 14.1) | Reference |  |  |
| Active Smoker | 49 | 4.96 (1.24 – 19.8) | 0.51 (0.12 – 2.17) | 0.361 | 0.346 |
| Ex-smoker | 81 | 4.51 (1.46 – 13.9) | 0.47 (0.14 – 1.57) | 0.221 | 0.190 |
| Medication (%) |  |  |  |  |  |
| No Medication | 12 | 6.78 (0.95 – 48.1) | Reference |  |  |
| Oral Medication | 430 | 8.31 (5.70 – 12.1) | 1.22 (0.18 – 8.20) | 0.836 | 0.810 |
| Oral Medication  +Insulin | 75 | 9.10 (4.09 – 20.3) | 1.44 (0.19 – 11.1) | 0.723 | 0.746 |
| Stroke and Cardiovascular Disease (%) |  |  |  |  |  |
| Absent | 22 | 8.54 (6.07 – 12.1) | Reference |  |  |
| Present | 495 | 5.24 (0.74 – 37.2) | 0.60 (0.08 – 4.56) | 0.622 | 0.546 |
| Gangrene (%) |  |  |  |  |  |
| Absent | 29 | 7.89 (5.51 – 11.3) | Reference |  |  |
| Present | 488 | 15.9 (5.98 – 42.5) | 2.58 (0.90 – 7.36) | 0.077 | 0.098 |
| Neuropathy (%) |  |  |  |  |  |
| Absent | 272 | 8.45 (5.18 – 13.8) | Reference |  |  |
| Present | 245 | 8.33 (5.25 – 13.2) | 0.96 (0.48 – 1.92) | 0.914 | 0.891 |
| Residence (%) |  |  |  |  |  |
| Urban | 185 | 4.49 (2.02 – 10.0) | Reference |  |  |
| Rural | 335 | 10.2 (7.04 – 14.8) | 2.50 (1.03 – 5.88) | **0.043** | **0.037** |
| P^1^: crude  P^2^: multivariate regression model adjusted by age, gender, diabetes duration, systolic blood pressure, fasting blood glucose, gangrene, body mass index (BMI), diabetes medication, and residential area. | | | | | |
